# Multi-omics analysis of histidine metabolism reveals FRZB-CRYM-associated cellular crosstalk in heart failure

**DOI:** 10.3389/fcvm.2026.1830967

**Published:** 2026-08-10

**Authors:** Songlin Xiao, Chunlei Mo, Qixing Liu, Li Xu

**Affiliations:** Department of Cardiovascular Medicine, People's Hospital of Tongchuan District, Dazhou, Sichuan Province, China

**Keywords:** cell communication, CRYM, FRZB, heart failure, histidine metabolism, single-cell RNA sequencing

## Abstract

**Background:**

The role of histidine metabolism in heart failure (HF), particularly its heterogeneous regulation across cardiac cell types, remains unclear. This study aims to define its mechanistic basis and identify key regulatory genes.

**Methods:**

We integrated multiple transcriptomic datasets, including four conventional and three single-cell RNA-seq (scRNA-seq) cohorts from the GEO database. A multi-step computational biology pipeline was employed, comprising gene set enrichment analysis (e.g., AUCell, ssGSEA), multidimensional machine learning (14 algorithms, e.g., LASSO, random forest), differential expression analysis, and cell-cell communication inference (CellChat, MultiNicheNet) to identify and validate core targets.

**Results:**

We identified significant activation of the histidine metabolism pathway in the heart failure (HF) microenvironment, particularly within fibroblast and myeloid cells. A high-histidine-metabolism macrophage subpopulation (GPNMB^+^) exhibited enriched energy metabolism and upregulated MHC-II signaling, while a corresponding fibroblast subpopulation (Fib_THY1) showed enhanced immune communication. From 23 candidates, a rigorous machine learning strategy pinpointed FRZB and CRYM as core targets. These genes were markedly upregulated in HF, demonstrated reliable diagnostic value (AUC: 0.87–0.98), and correlated strongly with histidine metabolism. Crucially, scRNA-seq revealed distinct cellular localization: CRYM in cardiomyocytes and FRZB in mesenchymal stromal cells. Cell communication analysis suggested a putative pathological positive feedback loop wherein FRZB^+^ stromal cells are predicted to influence metabolic reprogramming in cardiomyocytes. This study proposes a novel model of a “stroma-myocardium” feedback loop.

**Conclusion:**

This study systematically characterizes the potential role of histidine metabolism dysfunction in HF and supports a model of a putative “stroma-myocardium” positive feedback loop associated with FRZB (in stromal cells) and CRYM (in cardiomyocytes). These computational inferences position FRZB and CRYM as candidate biomarkers, while their regulatory axis provides a novel theoretical framework for potential therapeutic targets in HF.

## Introduction

Heart failure (HF) remains a critical and growing health burden worldwide, marked by steadily increasing rates of morbidity and mortality (1, 2). The pathophysiology of HF progression involves a multifaceted cascade of cellular and molecular alterations, central to which are pathological cardiac remodelling, chronic inflammation, excessive extracellular matrix (ECM) deposition leading to fibrosis, and profound disturbances in energy metabolism (3, 4). A key driver of both the initiation and advancement of HF is the dynamic remodelling of the cardiac microenvironment (CME). This microenvironment is composed of a diverse array of resident cells—such as cardiomyocytes, fibroblasts, and endothelial cells—along with infiltrating immune cells like macrophages and lymphocytes. Studies have shown that under HF conditions, specific pro-fibrotic fibroblast subpopulations become activated, accompanied by considerable infiltration of pro-inflammatory immune cells (5, 6). Yet, the computational models that initiate the activation of these distinct cell subsets and mediate their pathological interactions represent an important unresolved issue. In parallel, metabolic reprogramming has emerged as another fundamental characteristic of HF.

While the healthy heart depends mainly on fatty acid oxidation to meet its energy demands, in heart failure, cardiomyocytes shift their metabolic preference towards glycolysis—a shift reminiscent of the Warburg effect observed in other diseases (7, 8). Although glucose and lipid metabolic alterations have been extensively investigated, the contribution of specific amino acid metabolic pathways to cardiac remodelling in HF, particularly their cell-type-specific effects within the CME, is not well defined. Histidine metabolism, which sits at the intersection of energy production, inflammatory signalling, and oxidative stress responses, has not yet been thoroughly examined in the context of the failing heart's microenvironment. Advances in single-cell RNA sequencing (scRNA-seq), supported by increasingly powerful computational tools and machine learning (ML) methods, now provide unique opportunities to dissect—with high resolution—cellular diversity, metabolic states, and intercellular communication networks in the HF microenvironment (9, 10). In light of this, the present study is designed to apply an integrated multi-omics approach, combining scRNA-seq with bulk transcriptomic data, to systematically explore how histidine metabolism influences remodelling of the cardiac microenvironment in HF, and to identify and validate key genes and regulatory mechanisms involved.

## Materials and methods

### Transcriptome data acquisition

The data for this study were sourced from publicly available datasets. Bulk RNA-seq analysis incorporated four independent cohorts from the GEO database: GSE57338 (136 controls vs. 177 heart failure samples), GSE5406 (14 vs. 194), GSE141910 (166 vs. 200), GSE116250 (14 vs. 50) and GSE79962 (11 vs. 20) (10–14). For single-cell resolution, three scRNA-seq datasets were analyzed: GSE145154 (105,533 cells from 6 samples), GSE183852 (49,723 cells from 8 samples, including 2 controls and 5 HF cases), and GSE121893 (4,933 cells from 12 samples, including 2 controls and 10 HF cases) (15–17). Genes associated with histidine metabolism were identified using the corresponding pathway (map00340) from the KEGG database, with the full list provided in Supplementary Table 1.

### Transcriptome data processing

For bulk RNA-seq data, batch effects across different datasets were removed using the sva R package prior to machine learning analysis (18). Normalised expression matrices were employed, comprising FPKM (Fragments Per Kilobase of transcript per Million mapped reads) values for sequencing data and log2-transformed values for microarray data. For single-cell data, analysis was performed using the Seurat package (v5.30) (19). During initial quality control, we excluded cells with >5,000 or <200 detected genes, >10% mitochondrial gene content, and >5% ribosomal or haemoglobin gene content. The remaining cells were subsequently normalised using the SCTransform method. Principal Component Analysis (PCA) was performed based on the top 3,000 highly variable genes. Clustering was conducted using a resolution of 0.8, and cell types were manually annotated based on canonical marker genes. The Harmony algorithm was employed to integrate samples from different scRNA-seq datasets (20).

### Single-cell gene set enrichment scores

To evaluate pathway activity in individual cells, six different gene set enrichment approaches were applied: AddModuleScore, AUCell, UCell, singscore, ssGSEA, and GSVA. Using the Seurat platform, AddModuleScore computes a module score by contrasting the mean expression of a gene set against a randomly selected control set (with 100 genes). The AUCell tool (version 1.30.1) determines an Area Under the Curve value from the cumulative distribution of expression ranks within the gene set, which represents the global measure of pathway activity. In addition, it is specifically optimized for the high sparsity and dropout effect of single-cell RNA-seq data. Therefore, we use the enrichment scores calculated by this algorithm to perform subsequent analyses. UCell (version 2.12.0) follows a comparable logic but employs the Mann–Whitney *U* statistic to improve computational speed. With the singscore package (version 1.28.0), a score is produced that incorporates both gene ranking and expression level. It is worth noting that, due to the characteristic sparsity of single-cell RNA-seq data, the top highly variable genes were supplied as input for the ssGSEA and GSVA algorithms (as implemented in GSVA version 2.2.0) (21). For all subsequent analyses, the histidine metabolism score generated by AUCell was adopted as the main measure, while results from the remaining five techniques served for additional verification. The correlation analysis between AUCell scores and scores of other algorithms is shown in the Supplementary Table 2.

### Differential enrichment analysis

To identify functionally enriched categories, Over-Representation Analysis (ORA) was carried out with reference to the Gene Ontology (GO) database. The clusterProfiler R package (version 4.16.0) was employed to detect statistically over-represented terms associated with biological processes, molecular functions, and cellular components (22). Visualisation of the significant enrichment results was accomplished using ggplot2 (version 3.5.02). For the bulk RNA-seq data, differential expression analysis was performed with the limma package (version 3.62.1) (23). Genes meeting the thresholds of an absolute log2 fold change (|log2FC|) greater than 0.585 (corresponding to a fold change >1.5) and a *p*-value below 0.05 in comparisons between HF and Control samples were classified as differentially expressed.

### Machine learning model construction

After batch effect correction, the integrated dataset was divided for model construction. Given its large sample size, the GSE141910 cohort was assigned to the training set. The remaining three cohorts (GSE79962, GSE5406, and GSE57338) were set aside as external validation sets to evaluate the generalizability of the models. A diverse set of 15 machine learning algorithms was utilized, comprising: Neural Network, Logistic Regression, Linear Discriminant Analysis, Quadratic Discriminant Analysis, k-Nearest Neighbors, Decision Tree, Random Forest, Extreme Gradient Boosting, Ridge Regression, LASSO, Elastic Net, Support Vector Machine, Gradient Boosting Machine, Stepwise Logistic Regression, and Naïve Bayes. Following systematic optimization of hyperparameters, a total of 117 unique model variants were developed and assessed. Model performance was measured using four criteria: F1 Score, Area Under the Curve, Accuracy, and Recall. The resulting performance outcomes were visualized with the ComplexHeatmap package in R (version 2.24.1) (24).

### Intercellular communication analysis

Global cell-cell communication networks were inferred using the CellChat R package (version 2.1.2) (25). For analyses within macrophage and fibroblast subpopulations, CellChat objects were created separately for the “high histidine” and “low histidine” groups (defined by AUCell scores). CellChat infers communication probability based on a curated ligand-receptor (L-R) interaction database and the average gene expression within each cell cluster. The overall interaction strength and count were quantified and compared between the two metabolic states using the compareInteractions function. Differential signalling pathways and key “hub” populations (identified via netAnalysis_computeCentrality) were analysed to characterise the communication patterns specific to the high-histidine microenvironment. MultiNicheNet Analysis: To investigate specific, target-gene-level interactions between FRZB^+^ MSCs and CRYM^+^ cardiomyocytes, the MultiNicheNet R package (v2.0.1) was employed (26). This algorithm predicts L-R interactions correlated with downstream gene expression changes in the receiver cell. The analysis was conducted bidirectionally (MSC-to-cardiomyocytes and cardiomyocytes-to-MSC) comparing the HF versus Control conditions. MultiNicheNet prioritises active L-R pairs by assessing their correlation with the expression of predicted downstream target genes. Subsequently, the target genes regulated by these prioritised L-R pathways were analysed against the GO database to infer the biological consequences of the communication.

### Immune infiltration analysis

To quantify the abundance of key cell populations within the bulk tissue of the GSE141910 cohort, we employed the IOBR R package (version 0.99.8) (27). We specifically selected two complementary deconvolution algorithms encapsulated within IOBR to robustly assess the stromal origin of our candidate genes (e.g., FRZB): MCPcounter: This algorithm was chosen for its ability to provide abundance scores for specific cell types based on curated marker gene sets. Our analysis focused on the “Fibroblasts” score (Fibroblasts_MCPcounter) to test the direct correlation between our candidate genes and a specific mesenchymal cell population. ESTIMATE: In parallel, the ESTIMATE algorithm was used to calculate a global StromalScore. This score quantifies the overall presence of the entire stromal compartment (including, but not limited to, fibroblasts) within the tissue microenvironment. The rationale for this dual-method approach was to establish a more robust validation: by demonstrating that our candidate gene expression correlated positively with both a specific cell-type score (MCPcounter) and a global compartment score (ESTIMATE), we could more confidently confirm their dominant expression within the cardiac stromal lineage.

### Statistical analysis

All statistical analyses were conducted using the R (version 4.5.1) and Python (version 3.10) programming languages. Data comparisons across groups were performed using either an independent-samples *t*-test or a Kruskal–Wallis test. A *p*-value of less than 0.05 was regarded as statistically significant.

## Results

### Analysing cellular complexity in the cardiac microenvironment of HF versus Control groups via scRNA-seq

**Figure 1:**
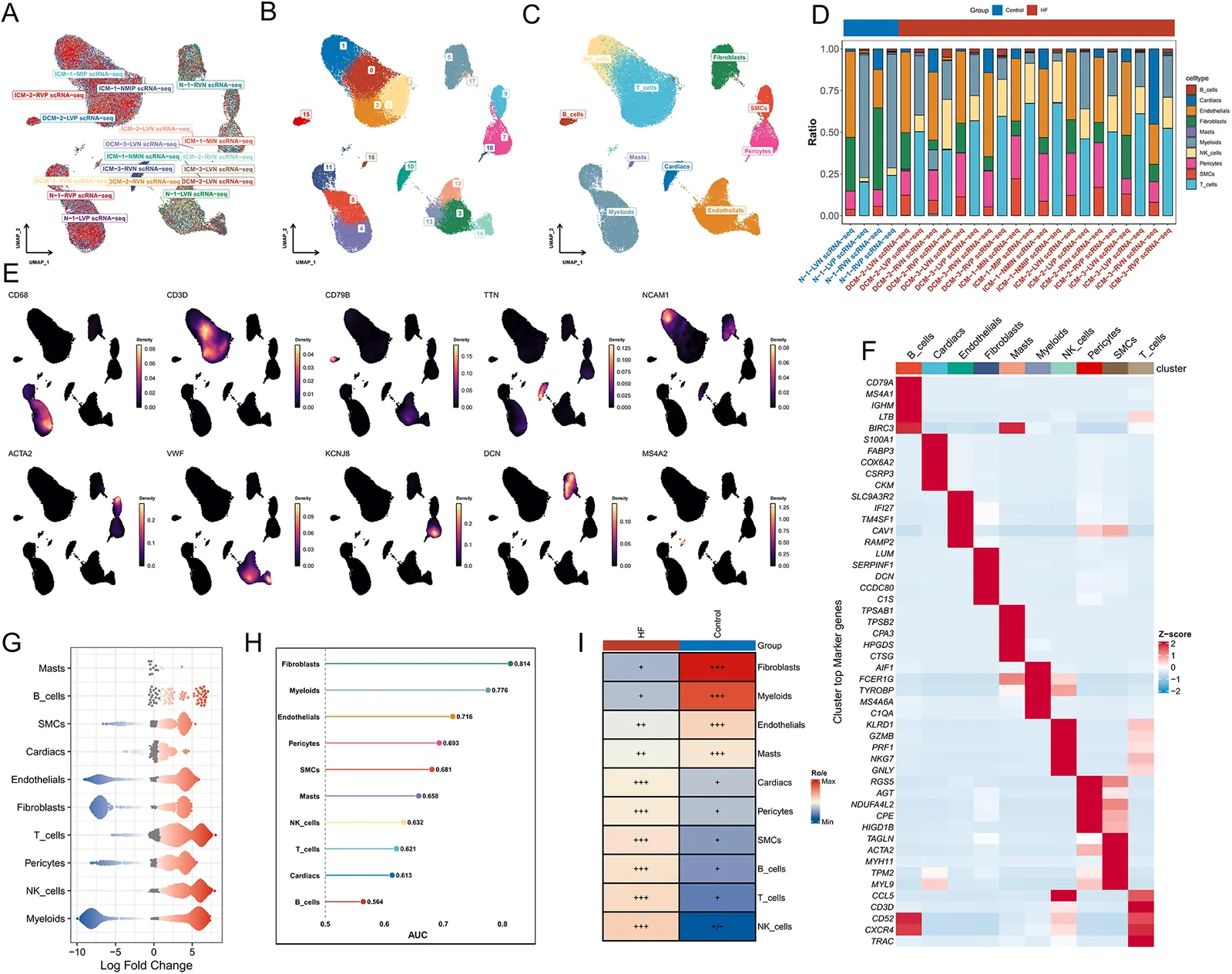
Single-cell heterogeneity analysis in HF and Control groups. **(A)** UMAP distribution of cells grouped by sample. **(B)** UMAP distribution of cells grouped by cluster. **(C)** UMAP distribution of cells grouped by cell type. **(D)** Proportional bar chart of cell types across samples and their subgroups. **(E)** UMAP plot of marker gene expression distribution by cell type. **(F)** Heatmap of differentially expressed genes by cell type. **(G)** MiloR analysis results by cell type. **(H)** Augur analysis results by cell type. **(I)** Observing cell type subpopulation preferences using RO/E.

This study examined remodeling of the cardiac microenvironment in heart failure (HF) through analysis of scRNA-seq dataset GSE145154. After implementing stringent quality control steps—such as filtering out low-quality cells (e.g., those exhibiting elevated expression of mitochondrial, erythrocyte, or ribosomal genes) and removing duplicates (Supplementary Figures S1A–C)—and integrating data from multiple samples (Figure 1A), all cells were grouped into 19 clusters via unsupervised clustering (Figure 1B) and assigned to 10 major cell types using established marker genes (Figure 1C). Comparative assessment of cellular composition uncovered notable shifts between HF and control samples (Figure 1D). Control samples were characterized by a higher abundance of stromal cells, including fibroblasts and endothelial cells, while HF specimens displayed pronounced accumulation of immune cells such as T cells and B cells, indicating substantial immune infiltration and tissue remodeling in HF. To confirm annotation accuracy, we visualized canonical marker gene expression on UMAP projections, which confirmed cluster-specific expression patterns (Figure 1E). Differentially expressed genes were also detected across individual cell types (Figure 1F).

To quantitatively evaluate compositional variation and intergroup heterogeneity, we applied three computational approaches. MiloR analysis (Figure 1G) identified significant enrichment of T cells, NK cells, pericytes, B cells, and SMCs in HF tissues. Conversely, myeloid cells, fibroblasts, endothelial cells, and cardiomyocytes were broadly distributed across both conditions, implying considerable heterogeneity within these populations. Augur analysis (Figure 1H), which evaluates transcriptomic state changes, highlighted fibroblasts and myeloid cells as the populations with the largest transcriptional shift between groups. Finally, subpopulation preference (RO/E) analysis (Figure 1I) further reinforced that fibroblasts and myeloid cells were more characteristic of control tissues, whereas lymphocyte subsets were preferentially associated with HF. Together, these complementary analytical approaches consistently identified fibroblasts and myeloid cells as the most heterogeneous and differentially distributed populations within the failing cardiac microenvironment. Based on these findings, we focused subsequent investigations on these two cell types.

### Multi-omics integrated analysis reveals heterogeneity in histidine metabolism within the HF cardiac microenvironment

**Figure 2:**
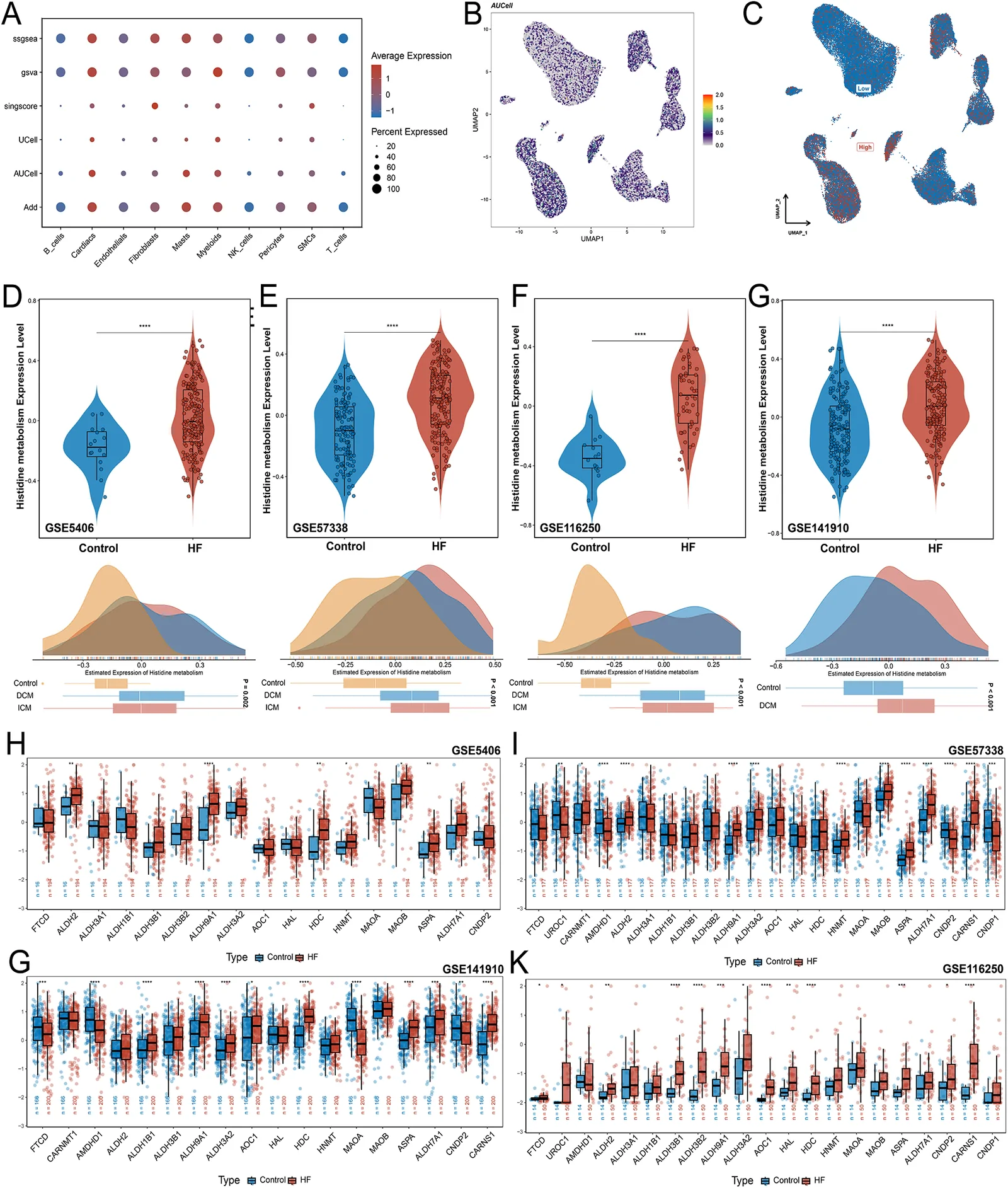
Heterogeneity of histidine metabolism in HF revealed by single-cell integrated conventional transcriptomics analysis. **(A)** Histidine metabolic activity observed across cell types under ssGSEA, GSVA, UCell, AUCell, singscore, and AddModuleScore algorithm conditions. **(B)** Expression of histidine metabolism on the single-cell UMAP plot. **(C)** Scoring groups for histidine metabolism in each cell predicted by the response model, with red indicating high groups and blue indicating low groups, grouped based on median total histidine metabolic activity. **(D–G)** Violin plots depicting differential expression of histidine metabolism between HF and Control groups across multiple datasets; ridge plots showing differential expression of histidine metabolism across Control, ICM, and DCM groups in multiple datasets (from left to right: datasets GSE5406, GSE57338, GSE116250, and GSE141910). **(H–K)** Box plots of differential expression of histidine metabolism-related genes between HF and Control groups (from left to right, datasets GSE5406, GSE57338, GSE141910, and GSE116250 respectively).

Based on the preceding findings, we next explored heterogeneity in specific biological functions within the heart failure (HF) microenvironment. Activity of the histidine metabolism pathway at single-cell resolution was evaluated by integrating six gene set enrichment algorithms—ssGSEA, GSVA, UCell, AUCell, singscore, and AddModuleScore. Among all cell types, cardiomyocytes, fibroblasts, mast cells, and myeloid cells displayed notably elevated histidine metabolism scores (Figure 2A). For a global characterization of the metabolic landscape, cells were stratified into high- and low-activity groups based on quartile values. This broad classification allowed for a comprehensive observation of the heterogeneous distribution pattern, where regions enriched for high-activity cells predominantly coincided with tissue zones associated with fibroblasts and myeloid cells (Figures 1I, 2B). To corroborate these findings at the tissue level, we analyzed four independent transcriptomic datasets (GSE5406, GSE57338, GSE141910, GSE116250). A complex dysregulated expression profile of histidine metabolism was detected in cardiac tissues from HF patients relative to controls (Figures 2D–G). Notably, this activation pattern was observed across HF patients with varying etiologies, including dilated cardiomyopathy (DCM) and ischemic cardiomyopathy (ICM), indicating that enhanced histidine metabolism represents a common biological hallmark of heart failure. Finally, we examined expression changes of key genes involved in this pathway. Rather than uniform upregulation, a dysregulated expression profile was observed (Figures 2H–K): several genes (e.g., ALDH2, HNMT, HDC) were significantly elevated in HF samples, whereas other critical genes (e.g., MAOA, CNDP1) showed marked downregulation.

### Analysis of histidine metabolism heterogeneity in myeloid cell subpopulations

**Figure 3:**
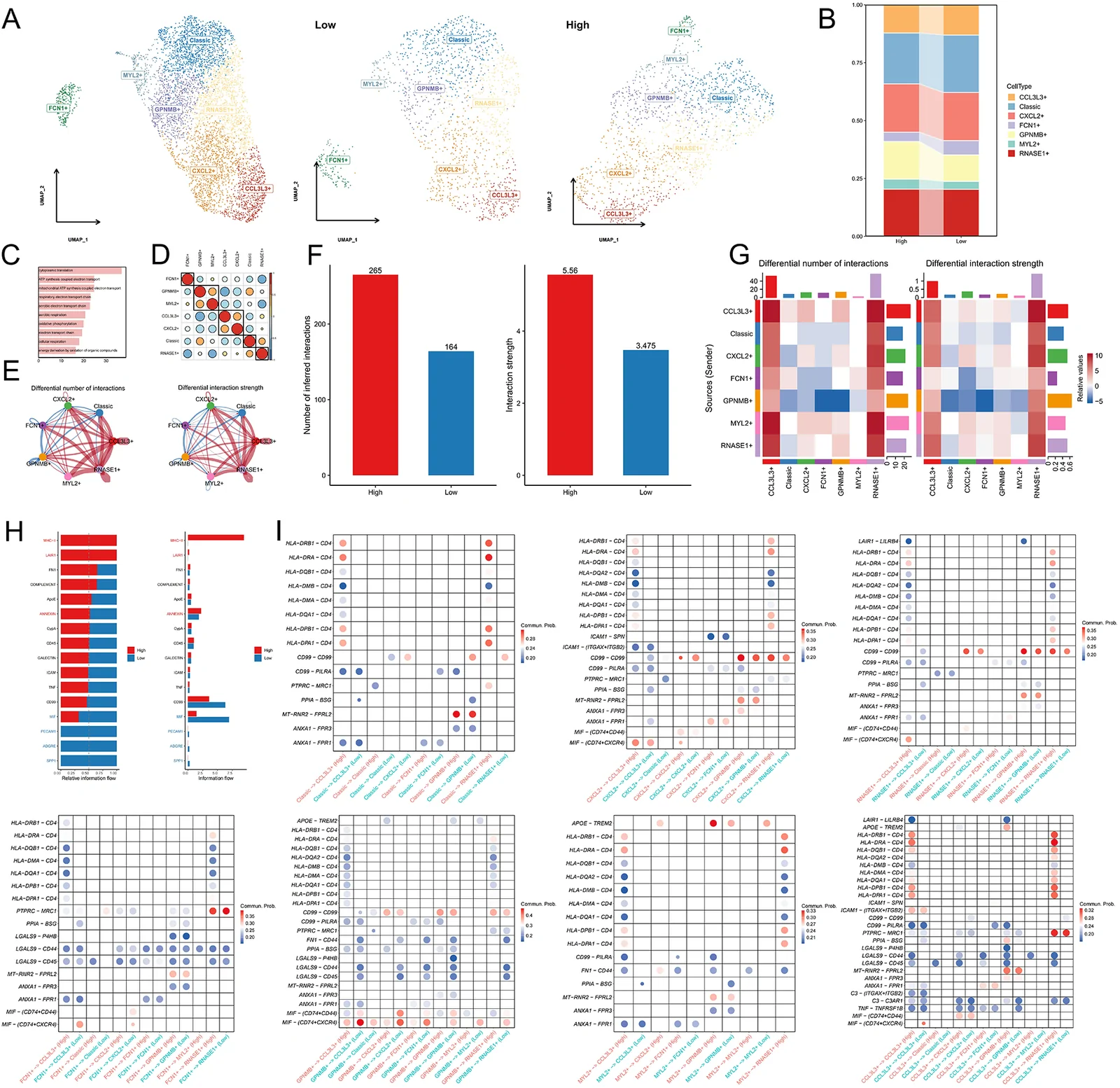
Subpopulation analysis of histidine metabolism in monocyte-derived macrophages. **(A)** All cells were divided into seven groups based on cluster-highly expressed genes, with UMAP distributions displayed (left panel). In the low histidine metabolism group, UMAP distributions were observed by cluster (middle panel). In the high histidine metabolism group, UMAP distributions were observed by cluster (right panel). **(B)** Proportional bar chart of subpopulation cells in high- and low-histidine groups. **(C)** GO enrichment analysis bar chart for the high-histidine group. **(D)** Correlation analysis between cell types (From blue to red, the correlation relationship increases successively). **(E)** Interaction patterns between cell types grouped by interaction strength (left panel) and interaction quantity (right panel) (red indicates enrichment specific to the high-histidine metabolic group; blue indicates enrichment specific to the low-histidine metabolic group). **(F)** Bar chart showing differences in interaction quantity (left panel) and interaction strength (right panel) between overall cells in high- and low-histidine metabolic groups. **(G)** Heatmap illustrating differences in interaction quantity (left panel) and interaction strength (right panel) between cell subtypes within high- and low-histidine metabolic groups (red indicates enrichment specific to the high-histidine metabolic group; blue indicates enrichment specific to the low-histidine metabolic group). **(H)** Display of differential ligands between high- and low-histidine groups (red indicates enrichment specific to the high-histidine metabolic group; blue indicates enrichment specific to the low-histidine metabolic group). **(I)** Differential ligand-receptor pairs and their interaction strengths between high- and low-histidine groups, grouped by cell subtype (red indicates enrichment specific to the high-histidine metabolic group; blue indicates enrichment specific to the low-histidine metabolic group).

To explore cellular heterogeneity in histidine metabolism during heart failure, we began by independently reclustering myeloid cells (Supplementary Figures S2A–C), classifying them into monocyte-macrophages (Mφ), neutrophils, and dendritic cells (DCs). Annotation accuracy was supported by subtype-specific marker gene expression, including LYVE1 in Mφ, FCER1A in DCs, and G0S2 in neutrophils (Supplementary Figure S2E). Among these, Mφ displayed the highest histidine metabolism activity (Supplementary Figure S2D), leading us to focus subsequent analysis on this population. Further subclustering of Mφ identified seven functionally distinct subtypes (Figure 3A): CCL3L3^+^, CXCL2^+^, GPNMB^+^, MYL2^+^, RNASE1^+^, FCN1^+^, and Classic macrophages, each showing clear transcriptional boundaries (Figure 3D) and defined by specific marker genes (Supplementary Figures S2F,G). We then applied CellChat to investigate communication patterns among macrophage subpopulations. The analysis revealed intensified interaction networks within “high histidine” macrophage clusters (Figures 3E–G), with CCL3L3^+^ and RNASE1^+^ subtypes serving as central signaling hubs. Differential pathway analysis further identified upregulation of MHC-II and LAIR1 signaling pathways in “high histidine” macrophages (Figure 3H). Notably, enhanced MHC-II signaling was observed across multiple subtypes within this group, especially when CCL3L3^+^ and Classic macrophages acted as signal senders (Figure 3I).

### Functional heterogeneity of fibroblast subpopulations and its association with histidine metabolism

**Figure 4:**
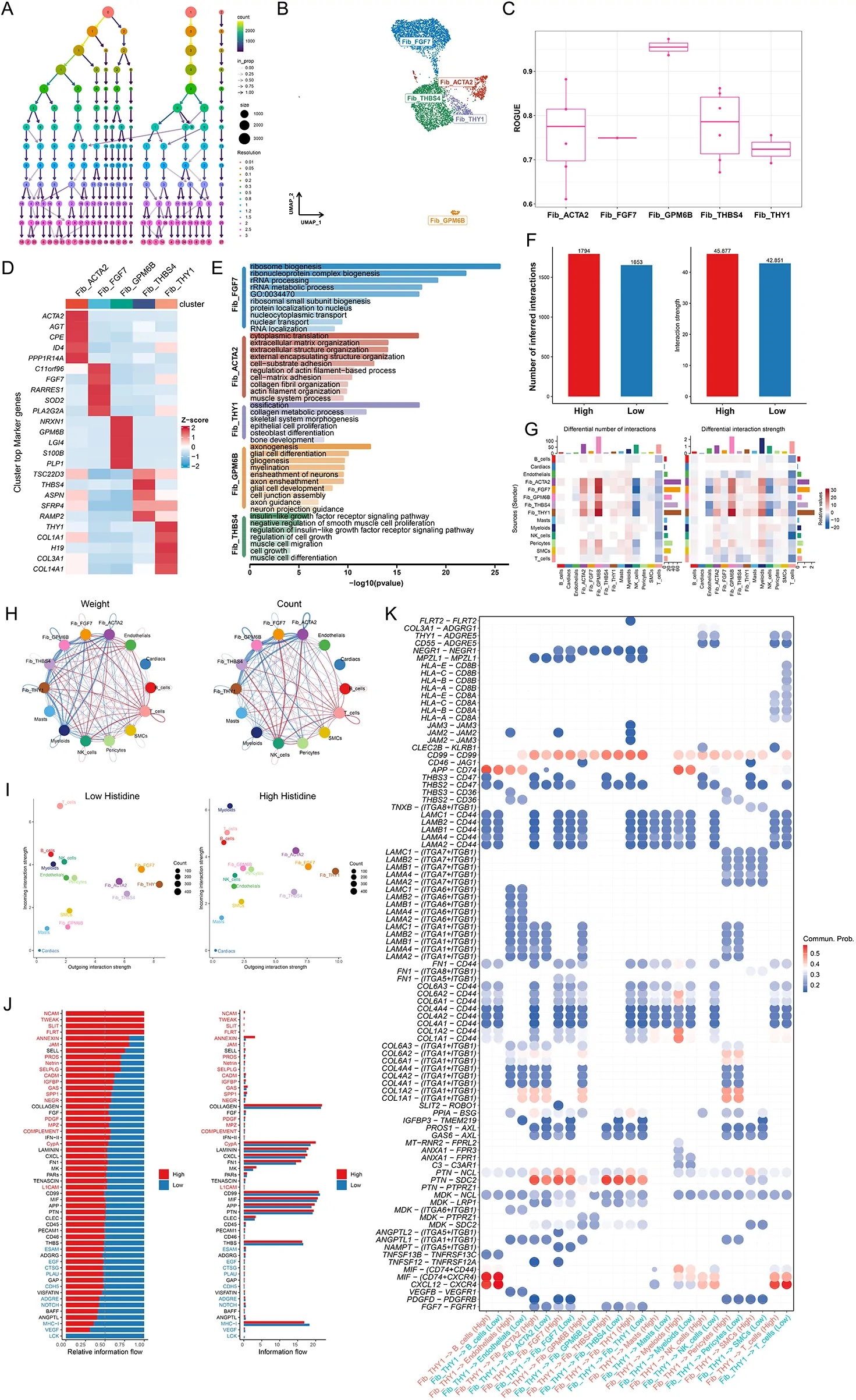
Subpopulation analysis of histidine metabolism in fibroblasts. **(A)** Dendrogram depicting fibroblast cluster distribution across varying resolution conditions. **(B)** UMAP distribution of cells grouped by clusters at 0.1 resolution. **(C)** RUGUE analysis results for cell subpopulations. **(D)** Heatmap of differentially expressed genes across subpopulations. **(E)** Subpopulation GO enrichment analysis bar chart. **(F)** Bar chart showing differences in interaction quantity (left) and interaction strength (right) between cells across high and low histidine metabolic groups. **(G)** Heatmap illustrating differences in interaction quantity (left) and interaction strength (right) between cell populations within high and low histidine metabolic groups. **(H)** Interaction patterns between cell types grouped by interaction quantity (left) and interaction strength (right). **(I)** Point plot of cell population input and output signal strengths between high and low histidine metabolic groups. **(J)** Display of differential ligands between high and low histidine groups. **(K)** Differential ligands observed between different histidine metabolic groups and other cell types, using Fib_THY1 as the ligand.

We next turned to fibroblasts to examine functional heterogeneity linked to histidine metabolism. After clustering at a stable resolution (0.1) (Figure 4A), five functionally distinct fibroblast subpopulations were identified (Figure 4B): Fib_ACTA2, Fib_FGF7, Fib_THBS4, Fib_THY1, and Fib_GPM6B. To assess the robustness of this classification, we first applied the ROGUE metric, which indicated high purity across all subtypes (ROGUE > 0.7), with Fib_GPM6B exceeding 0.9 (Figure 4C). We then performed differential gene expression analysis, which confirmed clear transcriptional distinctions among the five groups, aligning with their spatial segregation in UMAP visualization (Figure 4D). Functional characterization via Gene Ontology enrichment further highlighted specialized roles for each subtype (Figure 4E): Fib_FGF7 was associated with ribosome-related processes; Fib_ACTA2 was involved in extracellular matrix organization and cytoplasmic translation; Fib_THY1 was related to osteoblast development; Fib_GPM6B was connected to neuronal pathways; and Fib_THBS4 was enriched in insulin-like growth factor (IGF)-mediated signaling.

We then integrated these fibroblast subtypes with histidine metabolism activity profiles and analyzed their intercellular communication using CellChat. The results showed that fibroblast subpopulations with high histidine metabolism activity generally engaged in stronger cellular interactions (Figures 4F–I). Among them, the Fib_THY1 subtype emerged as the predominant signal sender within high-activity groups. Pathway-level examination identified several signaling routes—including those mediated by NCAM, TWEAK, SLIT, and ANNEXIN—that were markedly more active in high-histidine fibroblasts (Figure 4J). Focusing on interactions initiated by Fib_THY1, we observed a pronounced increase in signaling probability via the collagen-CD44 ligand–receptor pairs COL1A2-CD44 and COL1A1-CD44 between Fib_THY1 and myeloid cells in the high-histidine microenvironment (Figure 4K). Alterations in signaling patterns originating from other fibroblast subtypes are provided in Supplementary Figure S3.

### Single-cell and conventional transcriptome co-analysis for screening core histidine metabolism-related genes

**Figure 5:**
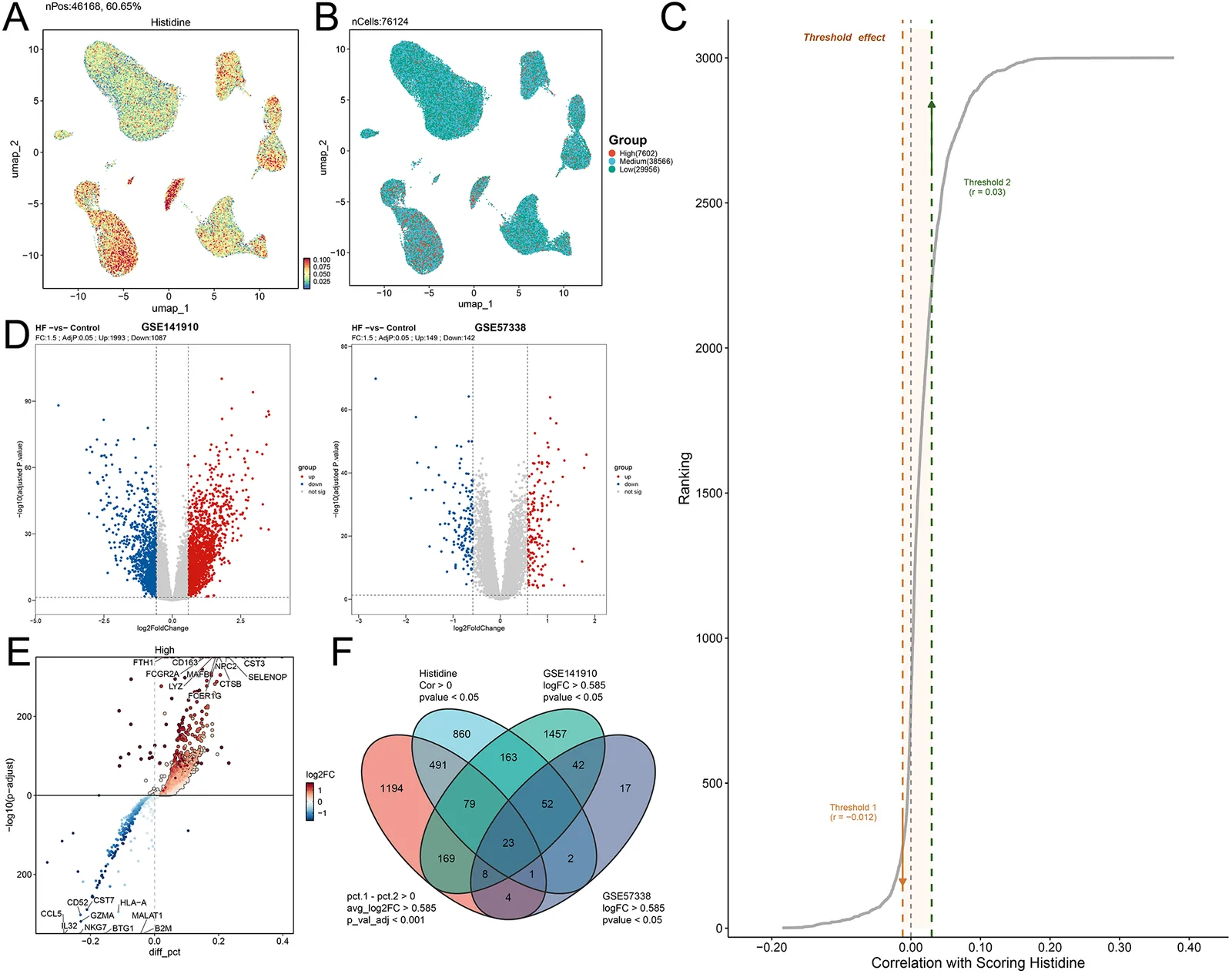
Screening of key histidine metabolism genes. **(A)** UMAP distribution of histidine metabolic activity. **(B)** Cell grouping based on histidine metabolic activity (cells with activity exceeding 90% of all cells classified as High group; cells with zero activity as Low group; remaining cells as Medium group). **(C)** Correlation analysis results between highly variable genes and histidine metabolic activity. **(D)** Differential gene screening using HF as positive control, with FC = 1.5 and AdjP < 0.05 as criteria (left panel represents GSE141910 dataset, right panel represents GSE57338 dataset). **(E)** Volcano plot displaying differences in high- and low-histidine metabolism groups. **(F)** Venn diagram illustrating intersecting genes.

To identify the most influential regulators with high precision, we implemented a more stringent, extreme-value filtering strategy for core target screening. Single-cell histidine metabolism pathway activity scores were determined (Figure 5A), enabling the classification of cells into three distinct categories: a high-activity group (top 10%, score > 90th percentile) to capture the most potent metabolic signatures, a low-activity group (absolute zero, score = 0) to serve as a clean baseline, and an intermediate group (Figure 5B). This rigorous contrast was designed to maximize the signal-to-noise ratio during the subsequent correlation and differential expression analyses. Subsequent Spearman correlation analysis between highly variable gene expression and metabolic activity scores revealed a sigmoidal relationship (Figure 5C), indicating a nonlinear association characterized by threshold behavior—gene expression initially increases gradually with rising pathway activity, then accelerates sharply beyond a critical point before plateauing under high-activity conditions. We applied a sequential filtering strategy to identify robust candidate genes: single-cell correlation analysis identified 1,671 genes positively correlated with metabolic scores (Cor > 0, *P* < 0.05); single-cell differential expression analysis revealed 1,969 genes significantly upregulated in high- versus low-activity groups (avg_log2FC > 0.585, pct.1 - pct.2 > 0, p_val_adj < 0.001; Figure 5E); conventional transcriptome validation using dataset GSE141910 identified 1,993 genes upregulated in HF versus controls (logFC > 0.585, *p* < 0.05; Figure 5D); and additional validation with dataset GSE57338 confirmed 150 differentially upregulated genes (logFC > 0.585, *p* < 0.05; Figure 5D). Intersection of these four gene sets yielded 23 core candidate genes (Figure 5F) that consistently demonstrated association with histidine metabolism activation in heart failure across both single-cell and bulk transcriptomic levels, establishing them as priority targets for subsequent investigation.

### Multidimensional machine learning screening for core target genes

**Figure 6:**
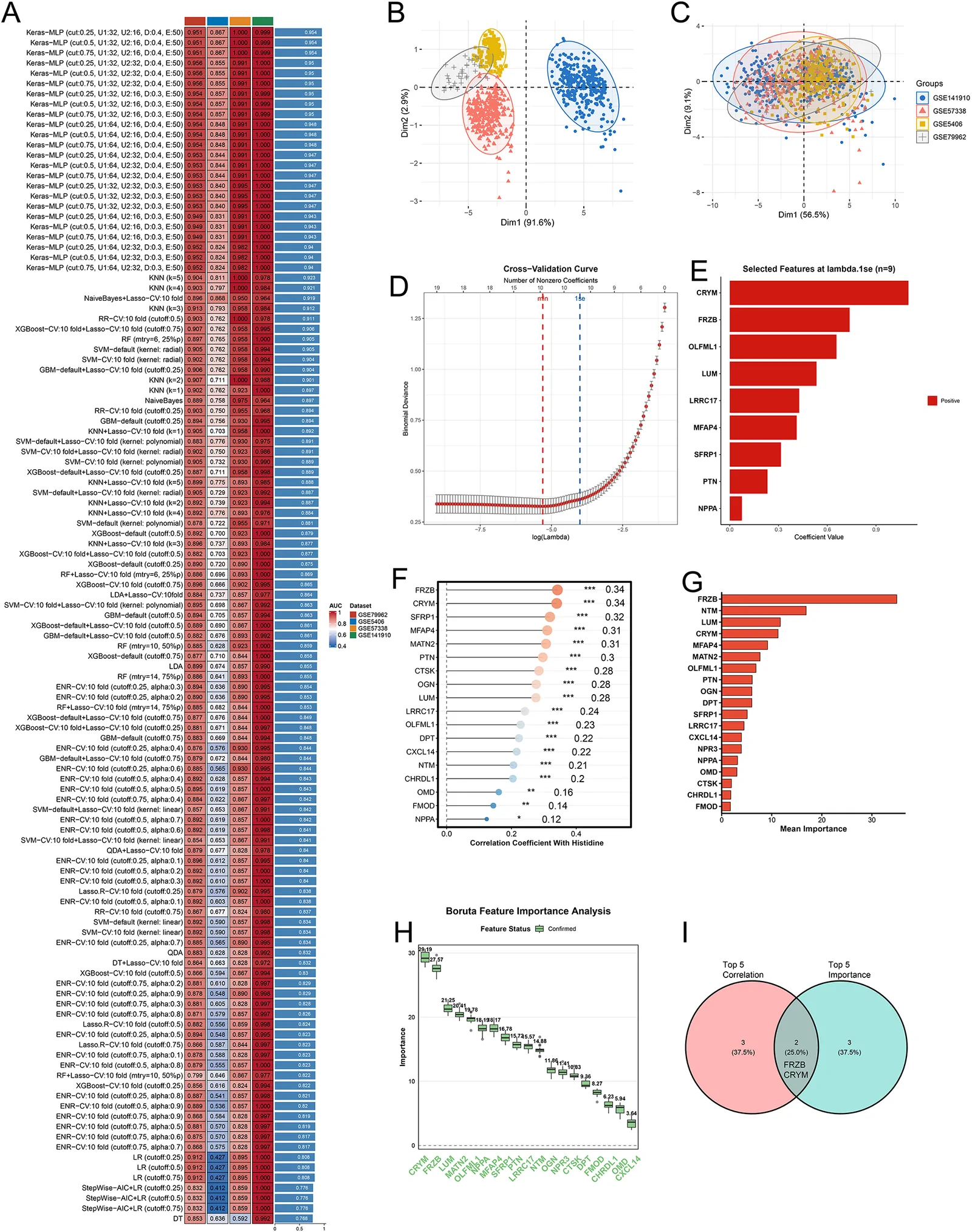
Multi-machine learning screening of candidate genes. **(A)** Diagnostic models established across four independent datasets using 117 binary machine learning algorithm combinations; heatmap displaying model AUC results. **(B)** PCA results prior to batch removal. **(C)** PCA results following batch removal. **(D)** Path dependence diagram of key genes obtained via LASSO analysis. **(E)** Observation of gene coefficients using lambda = 1se. **(F)** Bar chart of correlation analysis between key genes and histidine metabolic activity. **(G)** Statistical summary of variable importance across all model outputs, observing average variable importance of genes. **(H)** Boruta analysis of key genes. **(I)** Intersection of the top 5 genes most strongly correlated with histidine and the top 5 genes by variable importance ranking.

Building on the 23 candidate genes previously identified, we next sought to refine core targets and develop a diagnostic model. The four bulk transcriptomic datasets were initially harmonized through batch effect correction (Figures 6B,C). We then evaluated two gene sets for predictive performance: the full panel of 23 candidates, and a 9-gene subset refined by LASSO regression (using the Lambda.1se criterion) (Figure 6D). Both sets were used to train and optimize 15 machine learning algorithms—generating 117 model configurations—with performance assessed by AUC (Figure 6A), Recall, F1 Score, and Accuracy (Supplementary Figures S4A–C). Models incorporating all 23 genes exhibited strong diagnostic capability, reflected in high AUC values, reinforcing the relevance of histidine metabolism in heart failure and underscoring the collective importance of these genes. Interestingly, the 9-gene LASSO-derived subset did not surpass—and in some cases underperformed—the full gene set, particularly in non-linear classifiers such as Neural Networks and KNN. This implies that the original 23 genes form an information-rich, synergistic signature. Boruta analysis further validated this observation, confirming all 23 genes as “important” features (Figure 6H). As LASSO operates through linear constraints, it may have eliminated genes that contribute meaningfully in non-linear decision contexts. We therefore turned to machine learning-driven ranking to identify the most influential regulators among the 23 candidates. Two complementary ranking approaches were applied: at the transcriptomic level, we computed the correlation between gene expression and histidine metabolism activity scores (Figure 6F); separately, we extracted Variable Importance metrics derived from model outputs (Figure 6G). By intersecting genes ranked among the top five in both “correlation” and “model importance”, we identified two core target genes—FRZB and CRYM—following this multi-tiered validation.

### Validation of the diagnostic efficacy and expression of core target genes FRZB and CRYM across multiple cohorts

**Figure 7:**
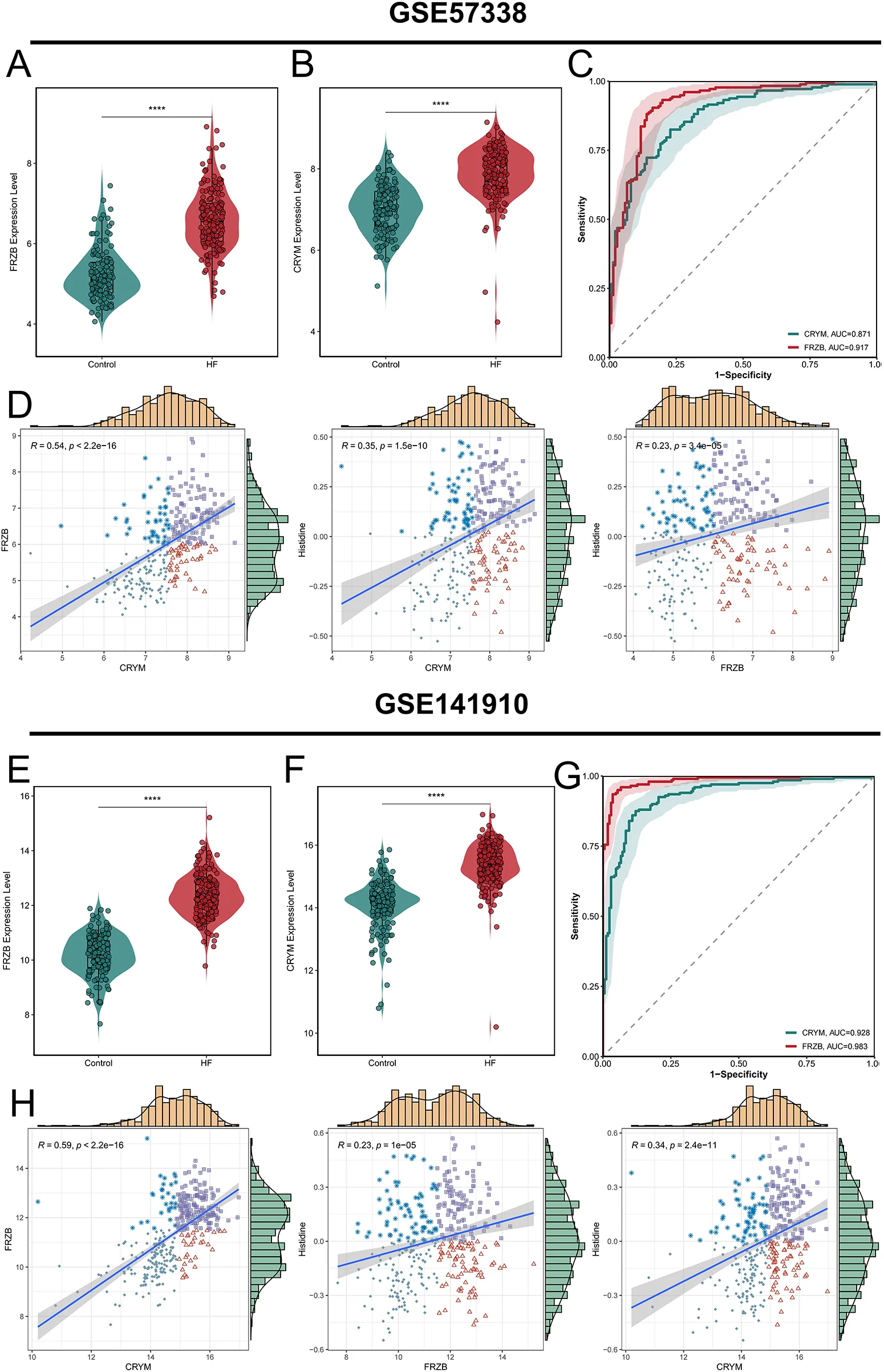
Expression validation of candidate genes. **(A)** Violin plot of differential expression of FRZB in HF versus Control groups within the GSE57338 dataset. **(B)** Violin plot of differential expression of CRYM in HF versus Control groups within the GSE57338 dataset. **(C)** Diagnostic ROC curves for FRZB and CRYM genes within the GSE57338 dataset. **(D)** Correlation scatter plot between FRZB, CRYM and histidine metabolism in the GSE57338 dataset. **(E)** Violin plot of differential expression of FRZB in HF versus Control groups from the GSE141910 dataset. **(F)** Violin plot of differential expression of CRYM in HF versus Control groups from the GSE141910 dataset. **(G)** Diagnostic ROC curve for FRZB and CRYM genes in the GSE141910 dataset. **(H)** Correlation scatter plot between FRZB, CRYM and histidine metabolism in the GSE141910 dataset.

To evaluate the clinical significance of the two core target genes (FRZB and CRYM), we examined their expression patterns and diagnostic performance in two independent bulk transcriptome cohorts (GSE57338 and GSE141910). In the GSE57338 dataset, expression levels of both FRZB and CRYM were significantly elevated in heart failure samples relative to controls (Figures 7A,B). Receiver operating characteristic (ROC) analysis demonstrated strong diagnostic ability, with area under the curve (AUC) values reaching 0.917 for FRZB and 0.871 for CRYM (Figure 7C). These results were consistently replicated in the GSE141910 cohort, where FRZB and CRYM again showed marked upregulation in HF specimens (Figures 7E,F) and achieved high diagnostic accuracy (AUC = 0.983 for FRZB; 0.928 for CRYM) (Figure 7G). Furthermore, we investigated the association between these genes and histidine metabolism pathway activity. Correlation analyses across both cohorts revealed two key findings: first, a strongly significant positive co-expression relationship between FRZB and CRYM (GSE57338: R = 0.54; GSE141910: R = 0.59; both *P* < 2.2 × 10^−^¹⁶), indicative of potential coregulation; and second, a consistent positive correlation between each gene and histidine metabolism scores (e.g., in GSE141910: FRZB R = 0.23, CRYM R = 0.34), reinforcing their roles as central drivers of histidine metabolic activation in HF. Notably, the current study focuses on transcriptome analysis at the organizational level. Further clinical validation in combination with the peripheral blood circulatory system (such as plasma or peripheral blood mononuclear cells) is required to evaluate its potential as a non - invasive biomarker.

### Single-cell expression localisation of core target genes FRZB and CRYM

**Figure 8:**
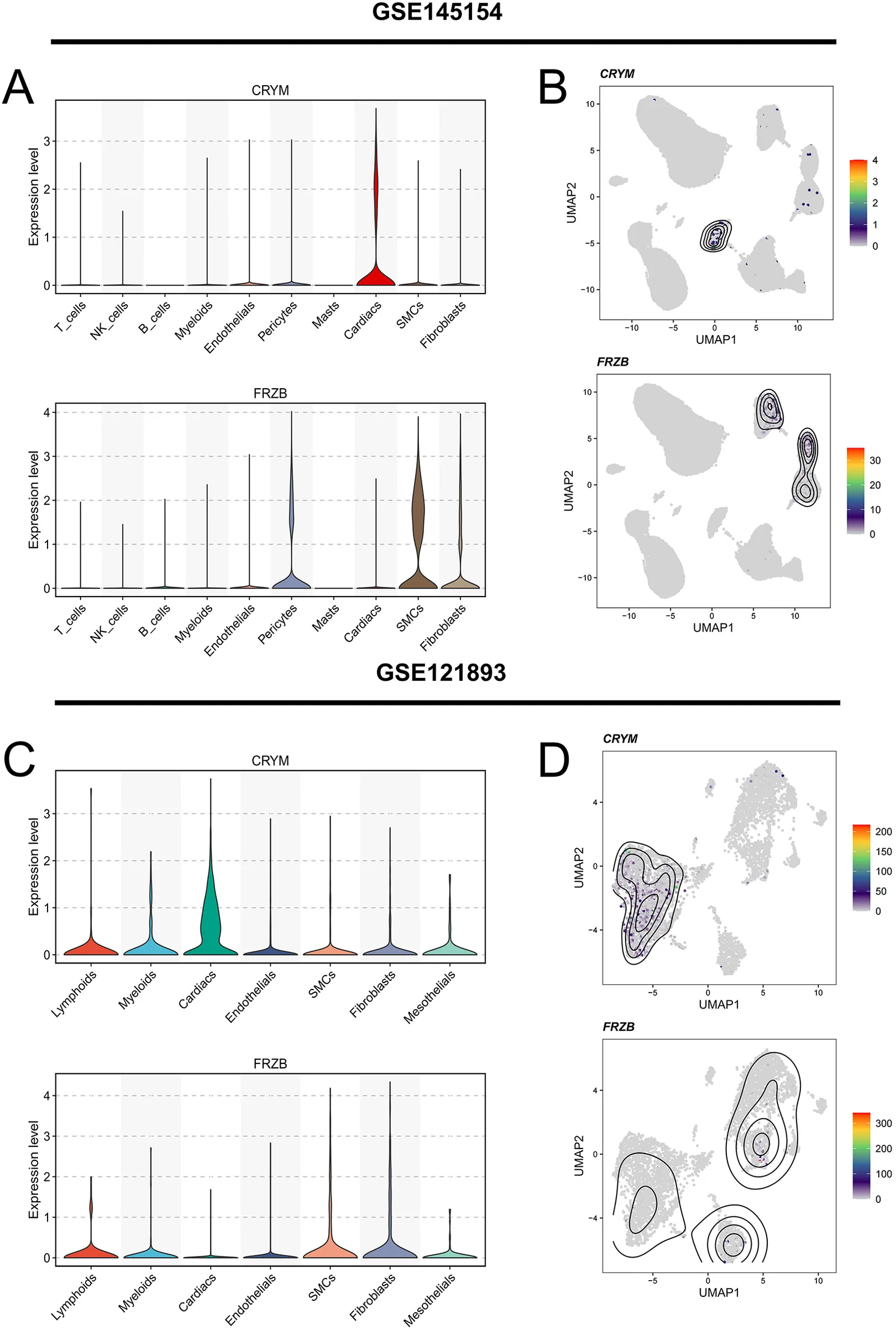
Expression localisation of candidate genes. **(A)** Violin plot of CRYM and FRZB differential expression across cell types in the GSE145154 dataset. **(B)** UMAP plots of CRYM and FRZB expression in the GSE145154 dataset. **(C)** Violin plots of differential expression of CRYM and FRZB across cell types in the GSE121893 dataset. **(D)** UMAP plots of CRYM and FRZB expression in the GSE121893 dataset.

To determine the cellular sources of FRZB and CRYM in the cardiac microenvironment, we mapped their expression patterns across three independent single-cell RNA sequencing datasets (GSE145154, GSE121893, GSE183852). The two genes displayed clearly distinct cellular expression profiles. CRYM showed highly specific localization, being predominantly and consistently expressed in cardiomyocytes in both the GSE145154 (Figures 8A,B) and GSE121893 (Figures 8C,D) datasets. This cardiomyocyte-specific expression was further supported by strong positive correlations between CRYM and canonical cardiomyocyte markers such as MYH6, MYH7, and TNNT2 (Supplementary Figure S5G). In contrast, FRZB was found to be primarily enriched within mesenchymal stromal lineages. In the GSE145154 dataset, it showed elevated expression in pericytes, fibroblasts, and smooth muscle cells (Figures 8A,B). This stromal preference was consistently observed in the GSE121893 (Figures 8C,D) and GSE183852 (Supplementary Figures S5A–D) datasets. To further confirm its stromal origin, we evaluated the relationship between FRZB expression and established stromal signatures, identifying strong positive correlations with both Fibroblasts_MCPcounter (cor = 0.42) and StromalScore_estimate (cor = 0.51) (Supplementary Figures S5E,F). In summary, the two core target genes display clear cellular lineage separation in the failing heart: CRYM is predominantly expressed by cardiomyocytes, while FRZB is mainly derived from mesenchymal stromal cells.

### Bidirectional communication between FRZB^+^ stromal cells and CRYM^+^ cardiomyocytes in the HF cardiac microenvironment

**Figure 9:**
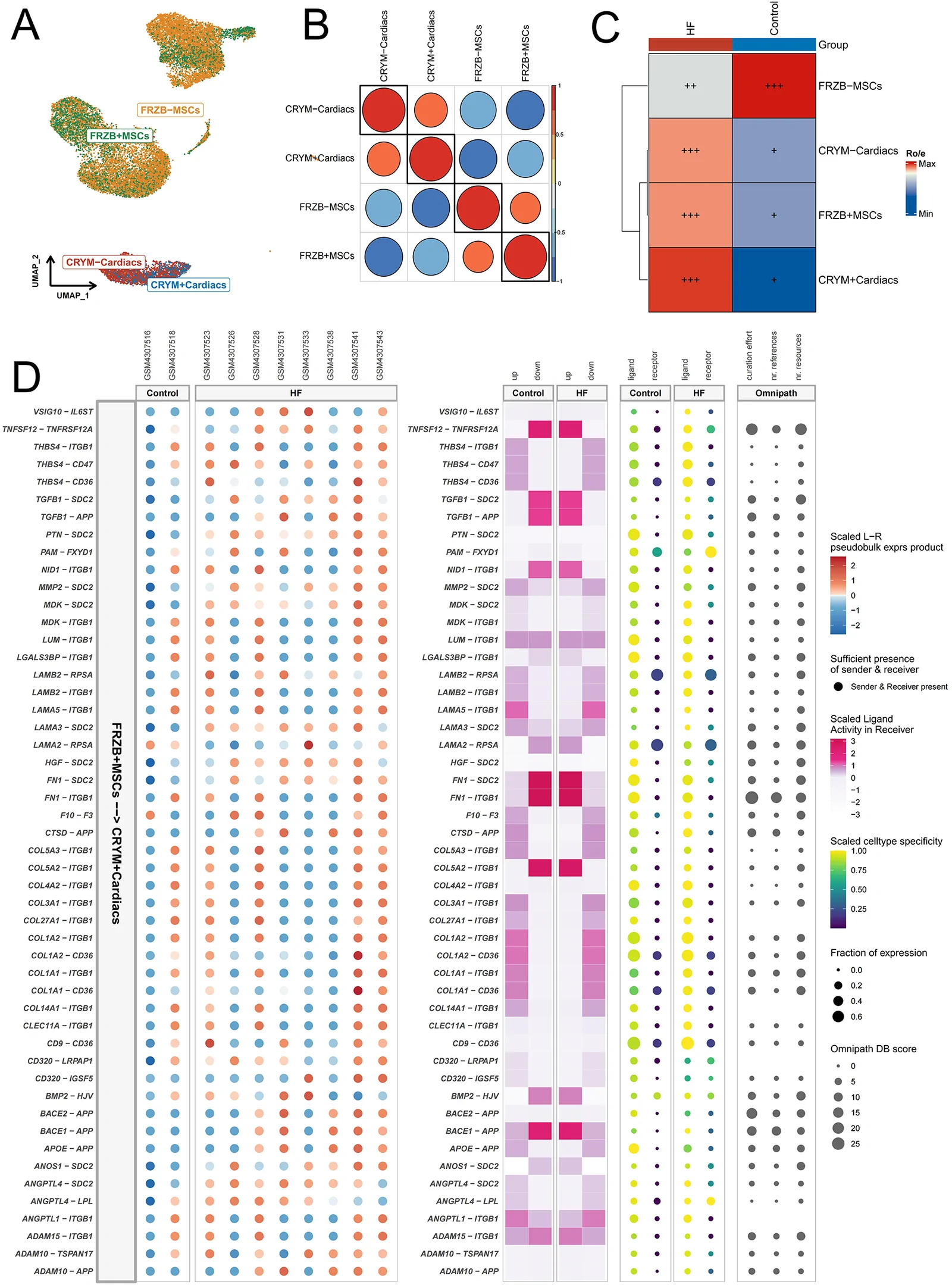
Interaction analysis between FRZB^+^ MSCs and CRYM^+^ cardiomyocytes cells. **(A)** All MSCs and cardiomyocytes were classified into FRZB^+^ MSCs, FRZB-MSCs, CRYM^+^ cardiomyocytes, and CRYM- cardiomyocytes based on whether the original expression counts of genes in the cells exceeded 1. **(B)** Correlation between cell populations. **(C)** Observation of tissue preferences among cell types. **(D)** Expression differences between donors (FRZB^+^ MSCs) and recipients (CRYM^+^ cardiomyocytes) were examined.

While our preliminary analysis identified Fib_THY1 as a key active fibroblast subpopulation, the molecular drivers connecting these stromal shifts to myocardial dysfunction remained elusive. Having identified FRZB (stromal-specific) and CRYM (cardiomyocyte-specific) as core metabolic regulators, we hypothesized that they mediate a critical stroma-myocardium communication axis that sustains HF progression. To investigate the functional specificity of cellular subpopulations expressing the core target genes in heart failure, we stratified mesenchymal cells (MSCs) and cardiomyocytes (cardiomyocytes) into four subsets according to FRZB and CRYM expression patterns: FRZB^+^ MSCs, FRZB^−^ MSCs, CRYM^+^ cardiomyocytes, and CRYM^−^ cardiomyocytes. UMAP projections (Figure 9A) and correlation analyses (Figure 9B) verified that these groups possess distinct molecular and spatial profiles. Tissue preference analysis (Figure 9C) further demonstrated that both FRZB^+^ MSCs and CRYM^+^ cardiomyocytes were preferentially localized in the HF microenvironment—in contrast to FRZB^−^ MSCs, which were more abundant in control tissues—with CRYM^+^ cardiomyocytes exhibiting the most prominent enrichment, indicating their potential role as central contributors to HF pathogenesis. We then utilized MultiNicheNet to examine communication between these enriched subpopulations, which revealed a strongly augmented bidirectional signaling network under HF conditions. In the stromal-to-myocardial direction (FRZB^+^ MSCs → CRYM^+^ cardiomyocytes), multiple ligand-receptor interactions—including FN1-ITGB1, FN1-SDC2, and TNFSF12-TNFRSF12A—were significantly elevated when FRZB^+^ MSCs served as signal sources (Figure 9D). Downstream target analysis (Supplementary Figure S6C) indicated that these signals primarily orchestrate metabolic rewiring in CRYM^+^ cardiomyocytes, affecting pathways such as nucleotide metabolism and glyoxylate/dicarboxylate metabolism. In the reciprocal myocardial-to-stromal direction (CRYM^+^ cardiomyocytes → FRZB^+^ MSCs), ligand-receptor axes such as LAMP4-ITGB1 and FGF7-NRP1 were also strengthened upon ligand emission from CRYM^+^ cardiomyocytes (Supplementary Figure S6A). Downstream enrichment (Supplementary Figure S6B) suggested that these cues from cardiomyocytes mainly provoke structural and pro-fibrotic phenotypic shifts in FRZB^+^ MSCs, including regulation of myocyte growth and actin filament-associated processes. Together, these observations support a computational model of a self-sustaining pathogenic cycle between FRZB^+^ stromal cells and CRYM^+^ cardiomyocytes in HF: stromal cells direct metabolic adaptation in cardiomyocytes, while cardiomyocytes reciprocally prime stromal cells for phenotypic transition, such as toward myofibroblast conversion. This mutually reinforcing dialogue appears to collectively propagate disease progression. Rather than a proven computational model, these inferred interactions suggest that stromal cells may contribute to metabolic adaptation.

## Discussion

At the outset, it is essential to clarify that all regulatory mechanisms, metabolic signatures, and communication axes proposed in this study are derived from computational inferences based on publicly available transcriptomic data. These findings represent in silico hypotheses that establish correlational associations rather than proven causality. Consequently, the observed activation of histidine metabolism and the proposed FRZB-CRYM feedback loop urgently await further experimental validation through cell co-culture systems, animal models (such as TAC or MI models), and clinical specimen analysis to confirm their biological and functional significance.

HF manifests as a multifaceted clinical condition defined by myocardial remodeling, dysregulated energy metabolism, and significant microenvironmental disruption (28). Emerging studies have confirmed that abnormal amino acid metabolism is closely involved in the occurrence and progression of heart failure, while the role of histidine metabolism remains insufficiently recognized in cardiovascular research. This investigation utilizes an integrated multi-omics framework, combining single-cell and bulk transcriptomic data, to systematically characterize histidine metabolism dysfunction as a key pathological feature in HF. We demonstrate that histidine metabolic dysregulation displays substantial cellular heterogeneity within the HF microenvironment, culminating in a pathological positive-feedback loop orchestrated by FRZB^+^ mesenchymal stromal cells (MSCs) and CRYM^+^ cardiomyocytes (CMs). Our initial characterization of the HF cardiac microenvironment revealed expected features including prominent immune infiltration and altered stromal cell proportions (17, 29). Extending beyond these established observations, computational analyses identified myeloid cells and fibroblasts as the most heterogeneous and transcriptionally dynamic populations in HF.

In fibroblasts, the high-histidine-metabolism Fib_THY1 subtype demonstrated enhanced signaling capacity, particularly through the COL1A-CD44 axis interacting with myeloid cells. As CD44 represents an established marker of fibrosis and inflammation, these observations imply that histidine metabolism additionally contributes to fibroblast activation and immune-stromal crosstalk (30, 31). A major methodological advancement involved implementing a rigorous screening strategy combining quadruple filtering with multidimensional machine learning to identify FRZB and CRYM have a strong statistical correlation with histidine metabolism pathway. Machine learning analysis confirmed the collective predictive strength of the 23-gene signature, retrospectively validating our screening approach. FRZB, a recognized Wnt signaling inhibitor expressed predominantly in stromal cells, has established roles in fibrotic processes across various tissues. While CRYM (μ-crystallin) has been implicated in NADH metabolism and thyroid hormone binding, its specific function in cardiomyocytes remains poorly defined. Our work provides the first evidence linking both genes to histidine metabolic dysregulation in HF, and precisely maps their cellular origins across three independent scRNA-seq datasets: FRZB predominantly localizes to stromal cells, whereas CRYM expression is primarily cardiomyocyte-specific.

FRZB (secreted frizzled-related protein 3) is a canonical antagonist of Wnt signaling that binds to Wnt ligands, thereby preventing their interaction with cell surface receptors (32). The Wnt/β-catenin signaling pathway is extensively implicated in cardiac fibrosis and pathological hypertrophy, and its inhibition has been shown to ameliorate adverse cardiac remodeling and improve function following myocardial ischemia (33). Beyond its established roles in skeletal development and osteoarthritis, FRZB has recently been linked to cardiovascular pathology. For example, reduced FRZB expression correlates with a synthetic vascular smooth muscle cell phenotype characterized by increased proliferation and inflammation (34). Furthermore, FRZB has been reported to exert protective effects against oxidative stress in failing hearts, although the precise molecular mechanisms (e.g., potential involvement of the AMPK/PGC-1α axis) remain to be fully elucidated (35, 36). In the context of heart failure (HF), FRZB has also been identified as an immune-related diagnostic gene in ischemic cardiomyopathy (35, 36). Nevertheless, the specific role of FRZB in cardiac stromal cells and its functional linkage to amino acid metabolism remain largely unexplored.

CRYM (μ-crystallin) encodes a NADPH-regulated thyroid hormone-binding protein belonging to the taxon-specific crystallin family (37). CRYM functions as a ketimine reductase with oxidoreductase activity, utilizing either NADH or NADPH as a cofactor (37). Beyond thyroid hormone binding—which carries important metabolic implications—CRYM modulates energy substrate utilization; available evidence suggests that its overexpression can promote a metabolic shift away from glycolysis toward increased fatty acid β-oxidation in certain tissues (38, 39). While CRYM expression has been linked to psychiatric, neuromuscular, and inflammatory diseases, its specific function in cardiac tissue has remained poorly defined (38, 39, 40). Our work provides the first evidence linking both FRZB and CRYM to histidine metabolic dysregulation in HF, and precisely maps their cellular origins across three independent scRNA-seq datasets: FRZB predominantly localizes to stromal cells, whereas CRYM expression is primarily cardiomyocyte-specific.

The most significant finding of this study is the identification of a pathological bidirectional communication circuit between FRZB^+^ MSCs and CRYM^+^ CMs. Both subpopulations show substantial enrichment in HF tissues. Under disease conditions, FRZB^+^ strzzzomal cells promote metabolic reprogramming (including nucleotide metabolism) in CRYM^+^ cardiomyocytes through signaling mediators such as fibronectin (FN1). This observation provides novel evidence that metabolic dysregulation in HF may be driven by stromal-derived signals. Crucially, CRYM^+^ cardiomyocytes are reciprocally associated with structural remodeling in FRZB^+^ stromal cells via factors including FGF7, with downstream enrichment in actin cytoskeleton pathways suggesting myofibroblast transdifferentiation. The computational inference of this “stroma-myocardium” positive feedback loop establishes a potential targetable axis, providing a novel theoretical framework for understanding HF pathogenesis. Our findings present considerable clinical implications. First, While FRZB and CRYM identify HF within the cardiac microenvironment with high sensitivity (AUC 0.87–0.98), their diagnostic potential in the peripheral circulation remains to be validated. Consequently, these markers are currently characterized as intra-cardiac pathological indicators. Second, the FRZB-CRYM regulatory axis and associated communication networks offer novel therapeutic targets. Unlike conventional single-target approaches, interventions directed at either FRZB or CRYM, or their downstream effectors (e.g., FN1-ITGB1 or FGF7-NRP1 signaling), may simultaneously address both core pathological processes in HF—metabolic reprogramming and fibrotic remodeling—potentially yielding superior treatment outcomes.

Several limitations warrant consideration. Our conclusions derive exclusively from bioinformatic analysis of public datasets, establishing correlations and predictions that require experimental validation. The observed histidine metabolism activation, FRZB/CRYM upregulation, and predicted cellular communication networks need confirmation through cell co-culture systems, animal models (e.g., TAC or MI models), and clinical specimen analysis. Furthermore, computational tools such as CellChat and MultiNicheNet infer interactions based on ligand-receptor expression, which may not fully capture the complexity of actual cell-cell communication. In summary, this multi-dimensional bioinformatic investigation systematically elucidates the heterogeneous role of histidine metabolism in the HF cardiac microenvironment, identifying FRZB and CRYM as core candidates strongly co-expressed with the histidine metabolic signature. The proposed pathological feedback loop between FRZB^+^ stromal cells and CRYM^+^ cardiomyocytes provides a novel theoretical framework for understanding HF pathogenesis. Future research should prioritize experimental validation of this circuit through: (1) FRZB or CRYM knockout in HF animal models to assess effects on cardiac metabolism and fibrosis; (2) co-culture systems to verify bidirectional stromal-cardiomyocyte regulation. These efforts will establish a foundation for developing novel HF therapies targeting histidine metabolism and the FRZB-CRYM axis.

## Conclusion

This comprehensive multi-omics study establishes histidine metabolism as a crucial, previously overlooked pathological component in heart failure. Moving beyond conventional pathway analysis, we demonstrate that this metabolic dysregulation constitutes a highly heterogeneous process coordinated by distinct yet interacting cellular populations. The central discovery of a pathological feedback loop between FRZB^+^ mesenchymal stromal cells and CRYM^+^ cardiomyocytes reveals a self-perpetuating computational model wherein stromal cells are associated with cardiomyocyte metabolic reprogramming, while cardiomyocytes reciprocally promote pathological stromal remodeling. These findings position FRZB and CRYM not only as reliable tissue-level markers of histidine metabolism dysregulation but as core components of a targetable axis, suggesting new therapeutic approaches focused on disrupting this detrimental cycle.

## Data Availability

The original contributions presented in the study are included in the article/Supplementary Material, further inquiries can be directed to the corresponding author.
